# Clustering and Analyzing Ensembles of Residue Interaction Networks from Molecular Dynamics Simulations

**DOI:** 10.1021/acs.jcim.5c01298

**Published:** 2025-10-03

**Authors:** Leon Franke, Christine Peter

**Affiliations:** Department of Chemistry, 26567 University of Konstanz, 78457 Konstanz, Germany

## Abstract

Network methods and molecular dynamics (MD) simulations have become essential tools for studying protein dynamics. However, applying network methods to MD simulations of flexible proteins is a major challenge, since the high conformational heterogeneity in such multistate systems can lead to vastly different network topologies across an ensemble. To address this, tools that can disentangle conformational ensembles on a network level are needed. Here, we propose a graph-based clustering framework that provides state-specific insight into the residue interactions of flexible proteins. The framework hinges on using the set of graph-theoretic closeness centralities of all amino acid residues as a structural fingerprint and input for unsupervised machine learning algorithms to perform dimensionality reduction and clustering. The resulting clusters—states with shared network topology—are subsequently fed back into the upstream workflow and characterized at every representation level. Based on the example of FAT10—a protein with intrinsically disordered regions and two folded domains connected by a flexible linker—we demonstrate how this approach can be used to understand the protein’s residue interactions on different, interconnected levels and to characterize its most populated states. Due to the modularity of the framework, it can be easily adapted, which makes it a suitable method to support network-based analyses of MD simulations for a wide variety of proteins.

## Graphical abstract

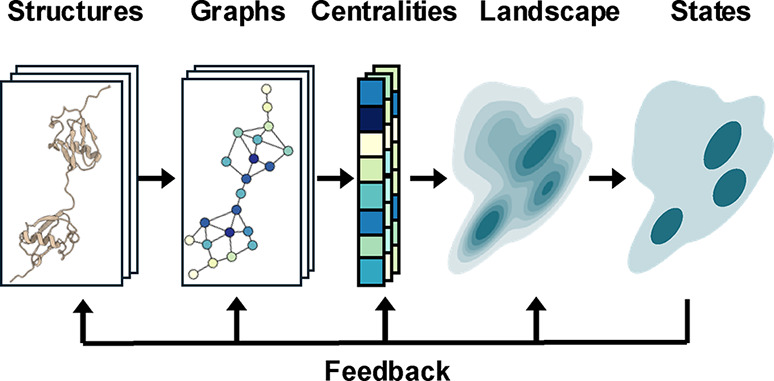

## Introduction

The three-dimensional structure and the function of proteins is governed by the interactions between the amino acids in their sequence. Residue interaction networks (RINs), sometimes also called protein contact networks (PCNs), cast these interactions into a network description, in which the amino acids are represented by nodes and the interactions or contacts are represented by edges.^1^ This simplified representation of the protein’s tertiary structure puts a focus on its contact topology and, applying tools from network theory, opens up many paths to gain insight into its function.^2–4^ There is a multitude of tools for constructing and analyzing RINs^5,6^ to investigate e.g. allosteric communication,^7,8^ the impact of mutations and system parameters,^9–11^ to identify important residues^12,13^ and interactions^14^ or to aid in protein engineering and design.^15,16^ While some of these tools construct the RIN from a single protein structure, others aggregate multiple protein structures into a network description of conformational ensembles.^17–19^ This is usually achieved by calculating a summary description across the entire ensemble, represented by edge weights in the summary network, e.g. based on contact frequency, correlation metrics or mutual information.^8,20,21^

Molecular dynamics (MD) simulations provide insights into the conformational ensembles of proteins and the conformational fluctuations at temporal and spatial resolutions that are difficult to access experimentally. With advances in simulation hardware and methods, ever-longer sampling times for ever-larger system sizes become computationally tractable.^22,23^ This makes it possible to model challenging biological processes and systems that require extensive sampling, such as proteins with multiple domains or intrinsically disordered regions.^24,25^ Typically, the corresponding ensembles are characterized by a high degree of conformational heterogeneity and multiple conformational states, which can substantially differ in their residue interaction topology. This makes it challenging to exploit the full potential of bringing together the dynamics data from MD simulations with traditional network methods. By aggregating the entirety of a conformational ensemble into a single network description, one may discard information on state-specific interactions,^8^ which can be crucial for protein function.^24^ One may even run the risk of misrepresenting interactions, e.g. by casting two mutually exclusive, sequentially occurring interactions into one cumulative network.

Unsupervised machine learning (ML) methods have been employed extensively to analyze conformational ensembles of proteins and disentangle them into separate states.^26–30^ In a typical workflow, each frame of the simulation trajectory is first translated into an appropriate numerical description or feature set based on the atom positions in the frame. Such a featurization can be done via a small, hand-crafted selection of system-specific low-dimensional descriptors or collective variables, such as radius of gyration (*R*_g_) or a count of native contacts. Alternatively, the feature set can be a relatively high-dimensional set of internal coordinates which are invariant to translation and rotation, e.g. dihedral angles or pairwise distances between amino acids. Such feature sets are too high-dimensional to usefully visualize or analyze directly.^31^ That is why, in the next step, dimensionality reduction algorithms are employed to find low-dimensional representations, also referred to as embeddings.^30,32,33^ Since the population of a given region in this low-dimensional representation of phase space is connected to the free energy in this region, phase space regions with high density correspond to (meta-)­stable conformational states of the protein. Consequently, density-based clustering algorithms are useful for grouping together conformations that are similar (close in embedding space) and belong to the same conformational state.^28,31,34^ With the appropriate choices for each step - featurization, dimensionality reduction, and clustering - such an ML workflow is a powerful framework to analyze and even enhance^35^ MD simulations, in particular for flexible protein systems.^25,36^

Combining ML algorithms for conformational clustering with protein network formalisms incorporates state-specific resolution to network analyses of heterogeneous protein ensembles. Here, we propose a framework to make this combination, centered around the closeness centralities as a graph-based node featurization. We construct a modular framework around this physically meaningful and interpretable feature set, using it as input for dimensionality reduction and clustering. A core tenet in the construction of the framework is the incorporation of a detailed characterization after clustering. The obtained clusters, interpreted as residue interaction states, are fed back through the workflow to understand inter- and intracluster relationships at each upstream representation level. The first step in this is visualizing the residue interaction landscape (i.e., the low-dimensional embedding) - which has been previously published^37^ - and using the visualization to contextualize the states found within it. Further upstream, graph representations of the network level and structure representations at the level of the simulation frames provide a state-specific, multilevel perspective on the discovered clusters. With this, the presented framework disentangles heterogeneous conformational ensembles into residue interaction states, resolving fine grained details of the interactions and enabling an informed selection of state representations for downstream network analyses.

We demonstrate the framework on a 30 μs MD data set of the two-domain, 165 residue signaling protein FAT10 (Human leukocyte antigen (HLA)-F adjacent transcript 10).^38^ Its conformational landscape is marked by high heterogeneity due to varying interactions between its N-terminal domain (ND) and its C-terminal domain (CD)^39^ and due to intrinsically disordered regions (IDRs).^40^ A major contributing factor to FAT10s cellular function as a signaling protein appears to be its flexibility, as the intrinsically disordered regions and the protein’s overall flexible fold aid substrate binding and degradation.^40^ Transient interactions between the two domains appear to play a role in stabilizing the protein, preventing it from denaturing.^39^ Yet, the interplay between its domains, the connecting flexible linker and the flexible loops is not yet fully elucidated. By applying the presented framework to FAT10 in its full length and to one domain in isolation, we analyze its conformational ensemble with a focus on residue interactions between the domains and within them. Characterizing the most prominent residue interaction states in both settings through the upstream representation levels of the workflow yields detailed insights on FAT10s domain interactions and its intrinsically disordered regions.

## Clustering and Analysis Framework for Ensembles of Residue Interaction Networks

Here, we outline the general framework to cluster and analyze ensembles of RINs as shown in Figure 1, more details and the concrete parameters used for the FAT10 example are presented in the Methods section. The workflow starts with calculating an RIN for each frame of the MD trajectory. In the RIN, amino acid residues are represented by nodes, and they are connected by an edge if they are in contact based on a geometric distance criterion. In the next step, a feature vector is calculated from every graph of the RIN assigning a number to each node that represents its role in the topology of the network. In the present case, the graph-theoretic metric of the closeness centrality is used. For each node, it is defined as the reciprocal of the mean shortest path length of that focal node *i* to all other nodes and is calculated using the following formula: $\frac{N}{\overset{N}{\underset{j}{\sum }}d_{ij}}$ , with *N* being the total number of nodes and *d*_*ij*_ being the shortest path length from node *i* to node *j*. In contrast with traditional contact maps, the closeness centrality encodes the information on the contact topology of the RINs in a compact, *N*-dimensional feature set, i.e. it scales linearly with protein sequence length rather than quadratically, which streamlines downstream analyses.^36^ It carries information on the global RIN topology as well as local information on the role of individual residues in the RIN,^13,41^ which makes it a highly interpretable feature set for RIN representation.^37^ The *N*-dimensional closeness fingerprints then serve as input for dimensionality reduction algorithms, which project the data into a low-dimensional embedding. Details on the closeness fingerprint for dimensionality reduction are discussed in Franke and Peter.^37^

**Figure 1:**
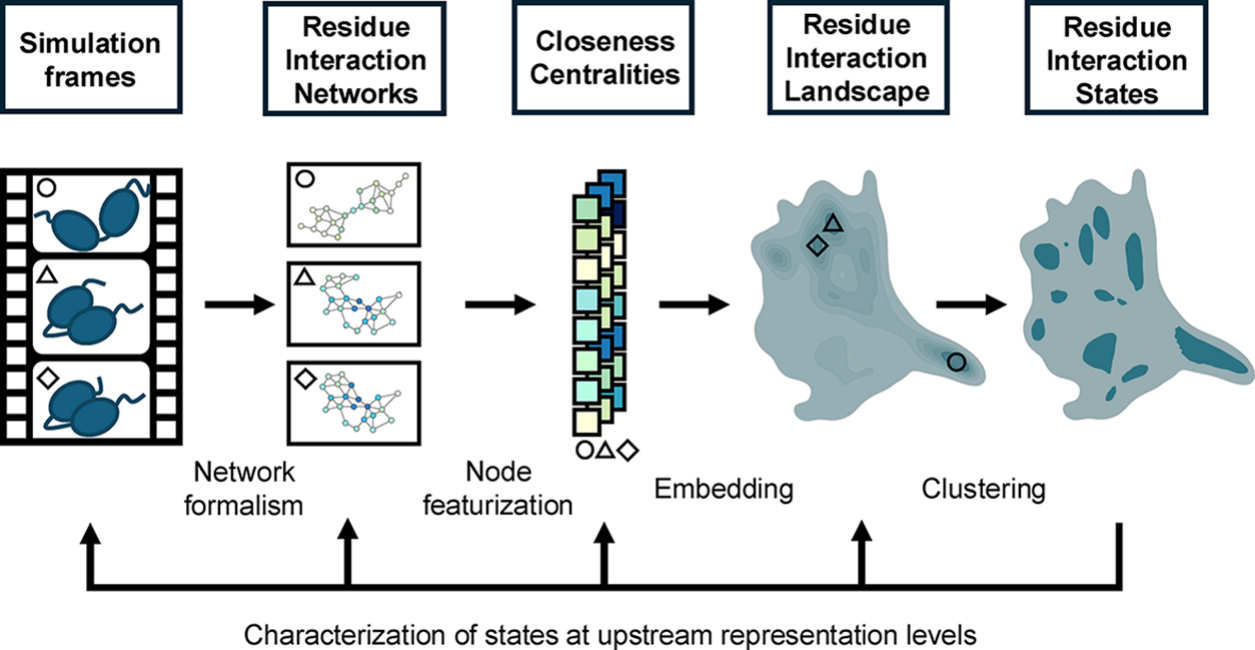
Framework for clustering and analyzing ensembles of RINs from MD simulations. After featurization, embedding, and clustering, the identified clusters (residue interaction states) are fed back into the workflow for characterization at upstream representation levels.

In this work, we show results for three different dimensionality reduction algorithms: Principal component analysis (PCA) is a linear dimensionality reduction algorithm^42^ while EncoderMap^43,44^ is a nonlinear method which combines the computational efficiency of a neural network autoencoder with a multidimensional-scaling like cost function. We also employ UMAP (Uniform manifold approximation and clustering)^45^ for a reduced portion of the data set, focusing on the CD of FAT10. In the next step, the low-dimensional map is clustered with a density-based clustering algorithm, here, we apply HDBSCAN (Hierarchical Density-Based Spatial Clustering for Applications with Noise).^46,47^ This allows the identification of high density clusters representing (meta-)­stable states^34^ in the residue interaction landscape, which we interpret as residue interaction states. A crucial step in the framework is the subsequent characterization of these states. By analyzing the cluster members at the different representation levels of the upstream workflow - the landscape, the closeness fingerprint, the RINs and the atomic structures - one can gain insight into the conformational ensemble of the protein, and understand its residue interaction states and the relationships between them.

## Results

In the following, we demonstrate for the example of FAT10 how the proposed framework can be advantageously applied to clustering the simulation data set into conformational states that can be further characterized with a spectrum of network analysis methods. We show how the closeness centrality can be used as an input for different dimensionality reduction algorithms and demonstrate (a) the modular nature of the framework and (b) that this gives access to information about the protein at different levels of resolution, putting a focus on either global, intermediate or local conformational behaviors. Furthermore, we show how such a workflow, which leads through different representation levels in a sequence of featurization steps, lends itself to network analysis of the simulation ensemble and its substates.

### Principal Component Analysis and Global State Characterization of FAT10

Extensive MD simulations of FAT10 resulted in a data set of 300,150 simulation frames. The atomic coordinates from these are then translated to RINs based on a distance criterion. From the RIN graphs, the closeness centrality for each node is calculated, resulting in a 165-dimensional closeness fingerprint for each frame. These fingerprints are used as input for a PCA in order to obtain a global overview of the conformational ensemble. We reduced the dimensionality of our closeness centrality data set from 165 to two by taking the first two principal components (PCs). Thus, we obtain a two-dimensional map to visualize the contact behavior of FAT10 in our simulation, i.e. a representation of FAT10s residue interaction landscape,^37^ where each of the simulation frames is assigned to a position in the map. The resulting map is shown in Figure 2. In Figure 2a, it is colored based on density, showing a compact, densely populated region on the right. Moving along PC1 (63% explained variance) toward the left, the map fans out, with several regions of different densities along PC2 (10% explained variance), among which the most densely populated region lies at the bottom left. The HDBSCAN clustering reflects this density distribution. The cluster outlines are overlaid on the PCA map in Figure 2b,c where the colorings of the map are chosen such that they illustrate the characteristics of the different regions and contextualize the clusters in the residue interaction landscape. The map in Figure 2b is colored based on the *R*_g_ of FAT10 and shows that PC1 is closely associated with the compactness of the molecule. A high *R*_g_ at the right of the map indicates that the molecule is mostly open, with no noncovalent contacts between its two domains. A low *R*_g_ means that the two domains have collapsed onto each other, forming different residue interactions at the domain interface. These differing domain interfaces are resolved along PC2. Figure 2c shows the PCA colored according to the domain–domain interface. The coloring is constructed by dividing the ND into two sections (red and blue) at residue Val43, counting the contacts of each section with the CD and subtracting blue from red. Red (high) values mean that the red section of the ND is in contact with the CD, blue (low) values mean that the blue section of the ND is in contact with the CD. The cluster outlines in the map show that HDBSCAN identifies the different residue interaction states, and the bundles of cluster representatives displayed in Figure 2b,c closely correspond to the characterization based on map colorings. A more detailed characterization becomes possible by going to the level of the closeness fingerprint where one can calculate the average closeness fingerprint for each cluster of interest. This helps to understand the distinct conformational characteristics for each cluster and illustrates the behavior of this feature set. In Figure 2d, the average closeness fingerprint is shown for the 3 most populated clusters. Cluster 0, with a high *R*_g_ and open structures has a low closeness centrality across all residues. This shows that the closeness centrality is sensitive to global conformational changes in a protein. When the two domains form contacts, all residues in the RIN are brought closer to each other via the bridging contacts, increasing the closeness centrality globally. Nonetheless, information on each individual residue is retained. Here, this can be seen by comparing the average closeness centralities of the two closed clusters 1 and 2. The residues with the highest centralities correspond to the residues forming the domain interface in the respective clusters. It is apparent that both clusters have high closeness centralities in different regions of the protein, indicating that they have distinct domain interfaces. This can also be seen in an overlay of the bundles of the cluster representatives for clusters 1 and 2 in Figure S1a in the Supporting Information. Such a global level of resolving conformational states - separating out a low-contact state and a few high-contact states - can give a good first impression of the residue interactions in a conformational ensemble. However, it may be necessary to have a finer separation of residue interaction states for investigating an ensemble in more detail. This can be achieved in different ways, such as clustering not only in two, but in several PCs or reclustering the existing clusters. It can also be achieved by applying nonlinear dimensionality reduction, which can improve the separation of the RINs in a low-dimensional embedding at the same dimensionality.

**Figure 2:**
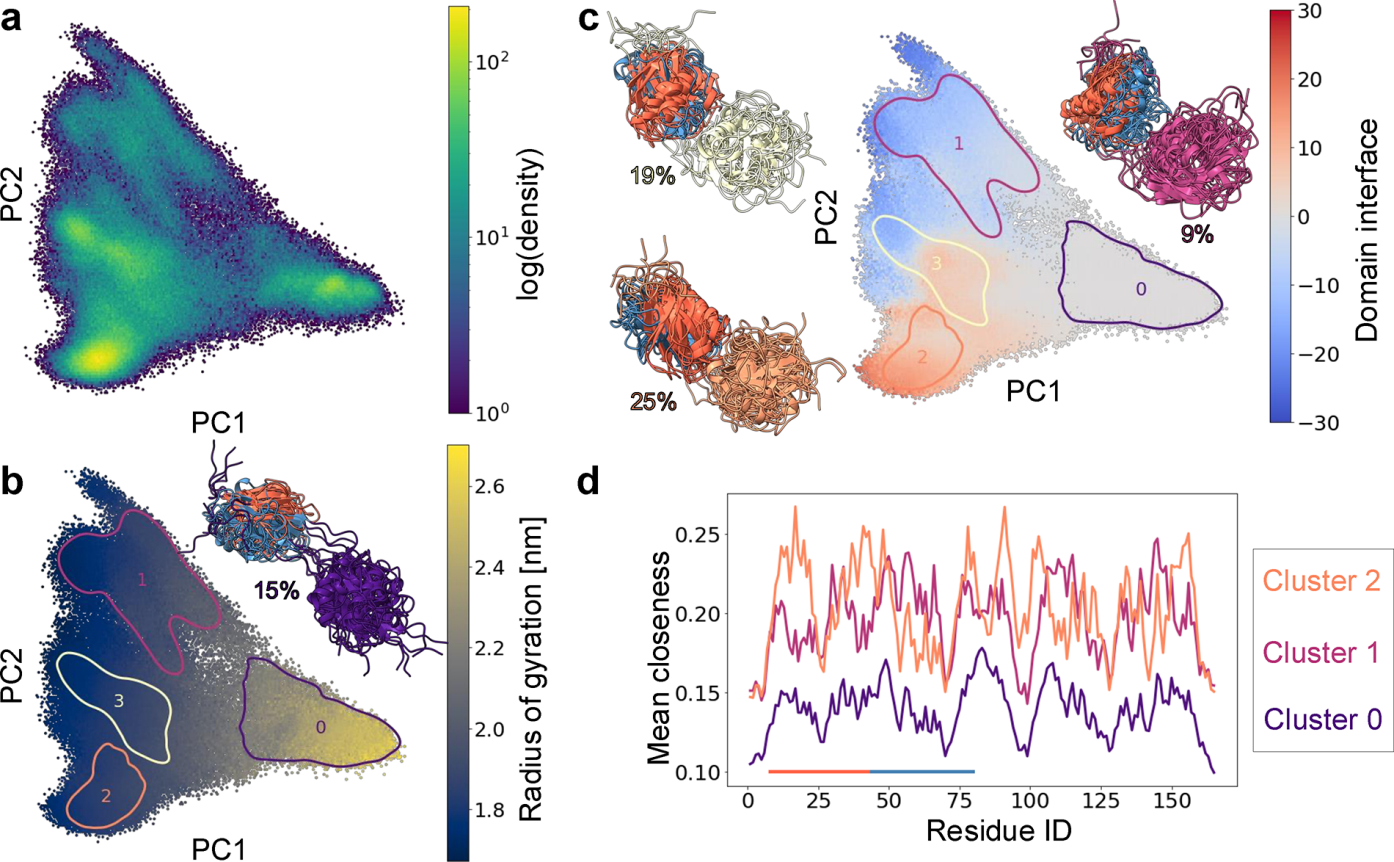
(a) PCA of the closeness fingerprints of FAT10 colored by log­(density). (b) PCA colored by radius of gyration overlaid with outlines of HDBSCAN clusters, inset: representative structures of cluster 0 (open cluster) with cluster population. (c) PCA colored by contact counts of domain interface overlaid with outlines of HDBSCAN clusters along with representative structures of clusters 1–3 (closed clusters) and cluster population. Domain interface coloring: Difference in contact counts (red means many contacts of the CD (cluster color) with the red section of the ND, blue means many contacts with the blue section, and gray means low contact count overall or equal contacts with red and blue). (d) Mean closeness fingerprints for clusters 0, 1, and 2, horizontal bar indicates sections of ND for contact counts.

### EncoderMap Analysis and Residue Interaction States of FAT10

We applied the nonlinear dimensionality reduction algorithm EncoderMap to the same 165-dimensional data set of closeness centralities of full-length FAT10 and generated a two-dimensional embedding displayed in Figure 3a,b. The map of the density in Figure 3b shows that the resulting embedding has a similar fan-like shape compared to the PCA. The map has a high density region at the bottom right and fans out toward the top left to resolve several other high density regions, which are classified as states by HDBSCAN (Figure 3a). With the appropriate parameter selection, HDBSCAN can detect several more distinct clusters in the EncoderMap than in the PCA. Interestingly, the EncoderMap does not have one single most highly populated closed state, but rather it has two: The clusters shown in red (R) and peach (P) in Figure 3a. It can be shown that these two states are collapsed into one cluster in the two-dimensional PCA (cluster 2 in Figure 2). They are separated along the third PC (shown in Figure S1b–d in the Supporting Information), which indicates that - given the same number of dimensions - the nonlinear EncoderMap is indeed capable of a finer separation than the linear PCA. If a central goal is visualization, a finer separation in fewer dimensions can be a useful feature. Nonetheless, it also shows that it is not necessarily always optimal to perform dimensionality reduction and clustering using only two dimensions. A characterization of the resulting clusters at the representation levels of the upstream workflow can give an indication of the suitability of the parameter selection for the clustering and point to potential adjustments. In addition, we can investigate characteristics of the individual residue interaction states. What do they have in common, what distinguishes them? At the level of the embedding, the closeness fingerprints, the RINs, and the atomic coordinates, this can yield insights into the conformational ensemble and inform the selection of workflow parameters. It can also guide the selection of cluster representations for downstream network analyses with state-specific resolution.

**Figure 3:**
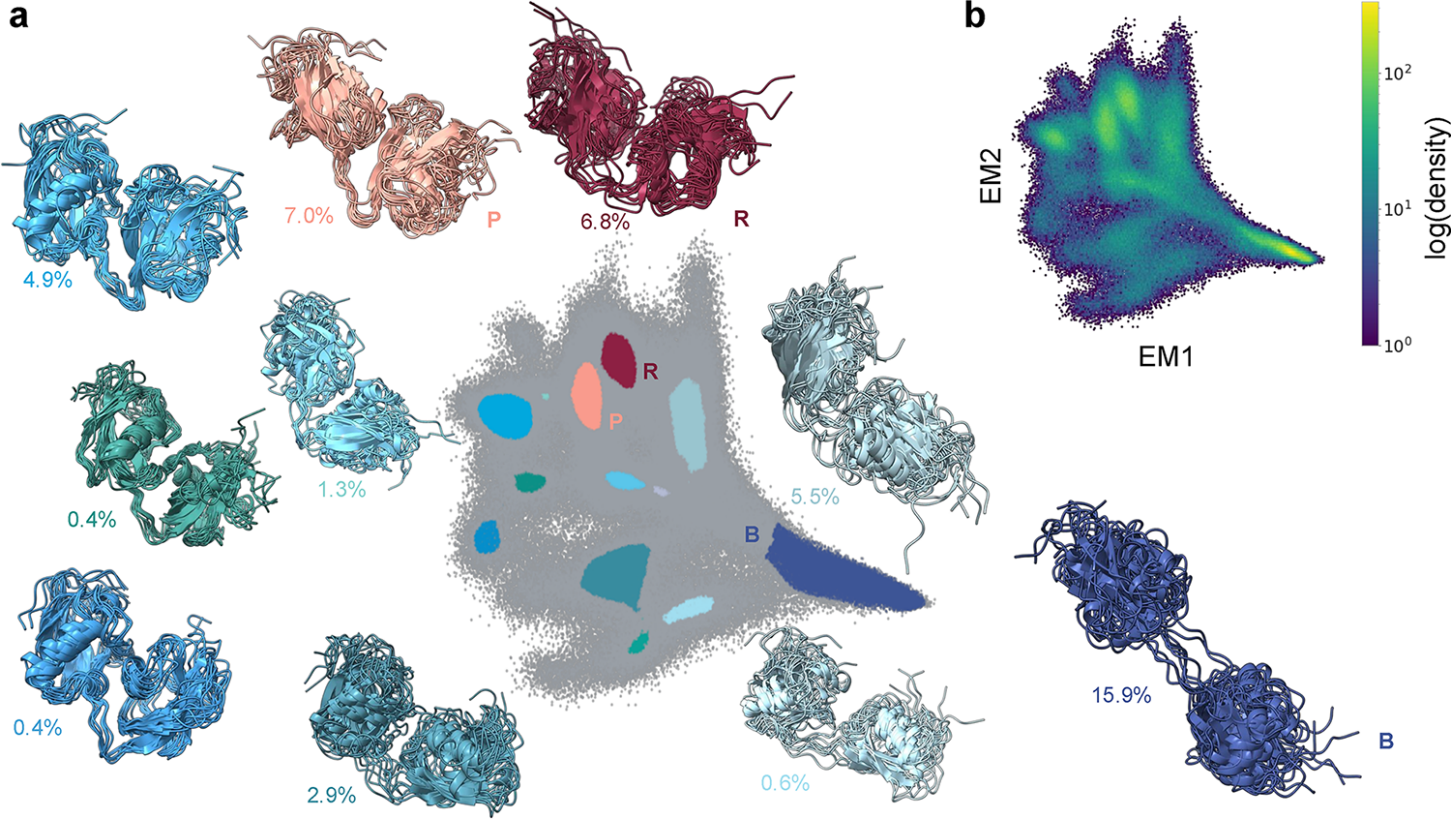
(a) EncoderMap of closeness fingerprints along with representative exemplar structures of HDBSCAN clusters and their populations, colored by cluster ID. For clarity, 3 clusters with population <0.2% were omitted. The three most populated clusters are labeled according to their coloring (blue (B), red (R), and peach (P)) to avoid confusion with the PCA clusters. Gray points are classified as noise. (b) EncoderMap of closeness fingerprint colored by log­(density).

Coloring the EncoderMap by *R*_g_ (Figure 4a) shows a separation of open and closed states similar to the PCA. The outlines of the most populated states (blue (B), red (R), peach (P)) are shown on the map to visualize their relative position in the embedding. Overlays of structures of the most populated EncoderMap states can be found in Figure S2a,b. Inspecting their average closeness fingerprints (Figure 4b) can give insight into similarities and differences between states on at residue-level resolution. We can see similarities to the PCA-based states: The open state (B) has a globally lower closeness and the red and peach closed states have high closeness centralities e.g. at residues Trp17 and Phe91, similarly to cluster 2 in the PCA (Figure 2d). It is also possible to identify residues that display differences: A closer look at the mean closeness fingerprints shows that the red and peach state are clearly distinct, e.g. by the large difference in closeness centrality for residue Lys152. We can pick out such residues with characteristic closeness centralities for specific states and visualize how the centrality of a residue changes across the residue interaction landscape. It is apparent that a high closeness centrality for Trp17 (Figure 4c) is a unifying feature for both states, but not for others in the map. In contrast, the closeness centrality for Lys152 (Figure 4d) is a feature fairly unique to the red state, distinguishing it from the peach state and other regions in the map. To understand this better, one can inspect the centroids of these clusters - i.e. a single structures that represent the cluster at the level of atomic coordinates (the selection of a centroid is explained in the Methods section). The centroids for the red and peach clusters are inset in Figure 4c,d and overlays of the centroids are shown in Figure S2c. The centroids illustrate the reason for the observations in the closeness fingerprint: Trp17 is a central part of the domain interface in both structures. It forms a π-cation interaction with Lys152 in the red cluster. In the peach cluster, this contact is not formed and Lys152 is not part of the domain interface. Rather, Trp17 forms hydrophobic contacts e.g. with Phe91. The two-dimensional map plays a crucial role in this evaluation as it provides an easily accessible visualization of residue-level features in the context of the global conformational behavior of the protein. This can also be seen when looking at EncoderMaps colored by the closeness centrality of other residues beyond those with the highest or most distinct closeness centralities. An overview of EncoderMaps colored by the closeness centralities of each residue of FAT10 can be found in Figure S3 of the Supporting Information. It shows how the role of a residue in the topology of the protein RIN can give it a characteristic closeness centrality signature across the residue interaction landscape. The two-dimensional map also gives context to understand how the RIN topology of the protein changes during the course of individual simulation trajectories (Figure S4). From the average closeness fingerprints of each cluster and from the cluster centroids, we can deduce whether FAT10s domains are in contact and which residues are part of the interface. We do not, however, get a full overview of the occurrence and frequency of the pairwise contacts specifically responsible for the domain interactions.

**Figure 4:**
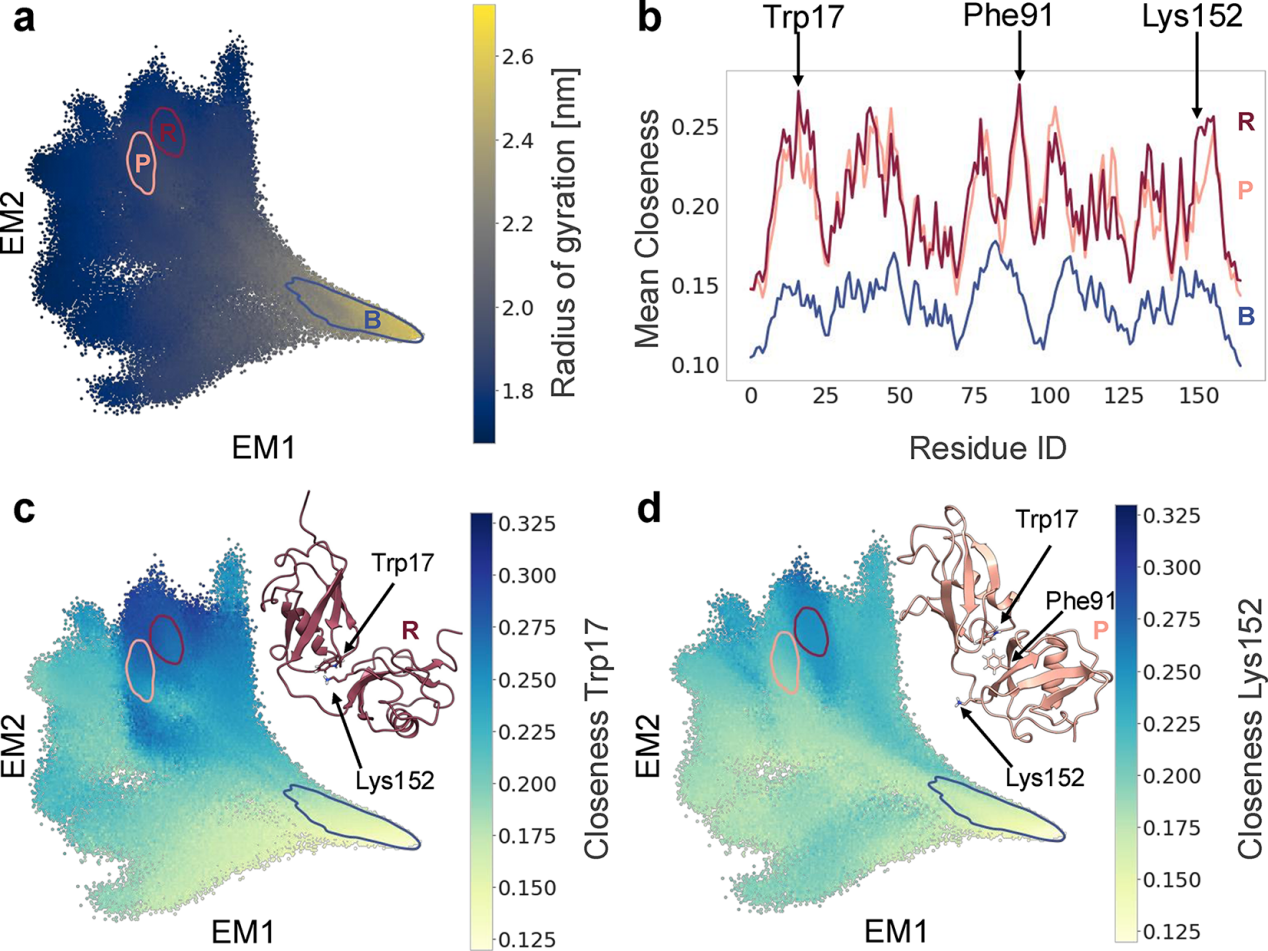
(a) EncoderMap of closeness fingerprints colored by *R*_g_ overlaid with outlines of the three most populated clusters (blue, red, and peach). (b) Mean closeness fingerprints for the most populated clusters. (c) EncoderMap of closeness fingerprints colored by closeness of Trp17, inset: centroid of red cluster, Trp17 and Lys152 are highlighted in stick representation. (d) EncoderMap of closeness fingerprints colored by closeness of Lys152, inset: centroid of peach cluster, Trp17, Phe91, and Lys152 are highlighted in stick representation.

For a representation of network topologies in the residue interaction states and to find shared and distinguishing contact motifs, we need to investigate the cluster members at the level of the RINs, i.e. the contact maps of the protein structures. For this, we propose the construction of a consensus graph^9^ for a cluster. This is done by calculating the probability of each contact in the cluster of interest and using them as weights for the edges of a weighted RIN representing the full cluster. Here, the edge weights are grouped into 3 categories (25–50%, 50–75% and >75%) for visualizing the consensus graphs using arc diagrams. For the red and peach cluster, the arc diagrams of the consensus graphs are shown in Figure 5a,b. The nodes of the graph are displayed along a straight line with arcs connecting them to represent the contacts or edges. The drawing style of the edge indicates the grouped contact probability in the cluster. The nodes are colored by the average closeness fingerprint in the cluster. In this instance, the intradomain contacts are removed from the diagram for clarity. This means the displayed arc diagram of a cluster shows how much the cluster members agree on a domain interface, both in terms of the specific contacts that are formed and the contact regions that are marked by an increased closeness centrality. The consensus graphs offer an RIN representation of the clusters and their visualizations give a detailed overview of the specific contact motifs that characterize a residue interaction state and how often they occur. The most likely contact for the red cluster is the contact between Trp17 and Lys152, which explains why the mean closeness centrality for those residues is so high in this cluster. Projecting the contact probability for a specific contact onto the EncoderMap embedding now allows us to understand where specific contacts of interest appear in the residue interaction landscape. Tracing the probability for the contact between Trp17 and Lys152 in Figure 5c, we can see that this domain contact appears only for conformations that have a low *R*_g_, i.e. that are already relatively collapsed. We can also see that this contact only appears in a very specific region of the residue interaction landscape, i.e. it is a contact motif that is unique to the red cluster. The consensus graph for the peach cluster (Figure 5d) shows that Trp17 also forms contacts with the other domain, but they individually have a lower prevalence and are formed with hydrophobic residues like the aforementioned Phe91. It should be noted that Trp17 and Lys152 are both located in flexible loops of the two interacting domains, whereas the hydrophobic residues interacting with Trp17 in the peach cluster are located in the β-sheets of the CD. The most likely contact for the peach cluster is between Ser84 and Glu87, with a probability of 91%. From the EncoderMap embedding colored by the probability of this contact, we can see that this tight turn in the linker (Figure 5d inset) is not only shared by the structures in the peach cluster, but by structures in other regions of the map as well. Importantly, this contact can form for very open structures with a high *R*_g_ and there are regions of the map in which this contact does not occur at all. Apparently, once this contact inside the linker has formed, only very specific domain interfaces are accessible for FAT10 as it closes, while other interfaces can no longer be formed. This illustrates that an aggregated network description not distinguishing between states would inadequately describe the domain interactions in the conformational ensemble of FAT10. The consensus graphs form a meaningful representation of the region of the conformational phase space associated with the respective cluster, and naturally describe the conformational subensemble with its distinct residue interactions. The interplay between the local interaction in the flexible linker and the global conformation of FAT10 is captured by investigating the network topologies with several state-specific consensus graphs and viewing them in the context of the residue interaction landscape for the full ensemble.

**Figure 5:**
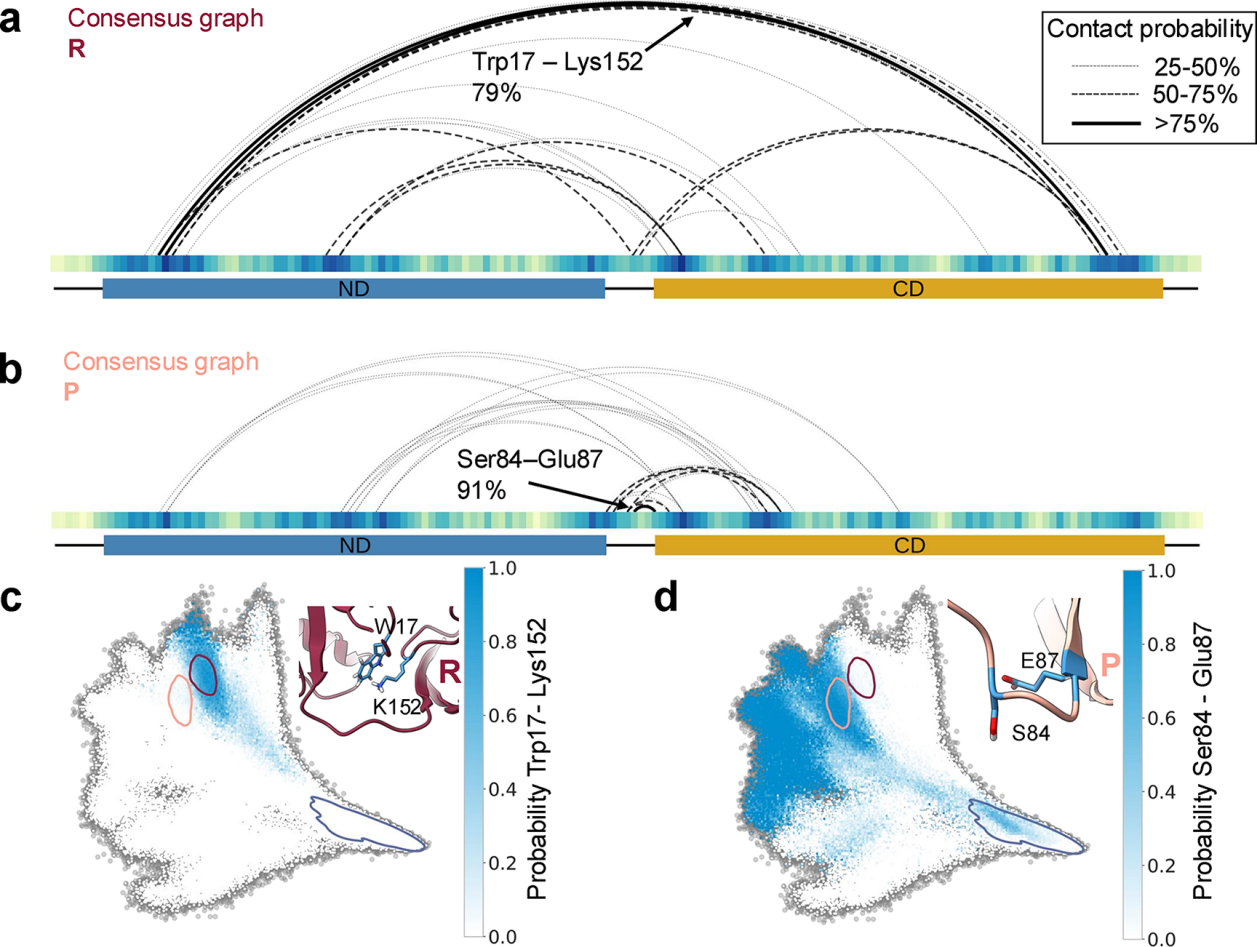
Arc diagrams of consensus graphs for the red (a) and the peach cluster (b). Residues are shown as tiles colored by the mean closeness fingerprint of the cluster. Contacts of the domain interface are shown as arcs with a drawing style based on the contact probability in the cluster. The most likely contact for each cluster is highlighted. Contacts within the domains are omitted for clarity. (c) EncoderMap colored by probability of contact Trp17-Lys152. Inset: centroid of R, Trp17, and Lys152 are highlighted as blue sticks. (d) EncoderMap colored by probability of contact Ser84-Glu87. Inset: centroid of P, Ser84, and Glu87 are highlighted as blue sticks.

### UMAP Analysis of Residue-Level Conformational Changes in the C-Terminal Domain of FAT10

While a perspective connecting global and local levels can be insightful in particular for highly flexible protein systems, there may also be relevant local changes of residue interaction networks, e.g. within globular proteins or domains. Here, we are investigating the globular C-terminal domain of FAT10 in isolation. The MD simulation trajectories are from the data set investigated above. However, the network construction is done only for the CD (residues 88–159), omitting contacts formed with the ND. The 72-dimensional closeness fingerprints from this analysis are input into the dimensionality reduction algorithm UMAP, which bases its embedding on local distances in embedding space, thus also resolving local behaviors better - potentially at the cost of losing global information.^42^ The UMAP embedding of the closeness fingerprints of the CD is shown in Figure 6a, colored by density. The results of the HDBSCAN clustering are shown in Figure 6b. With the chosen setting, HDBSCAN separates the map into several clusters. Depending on the parameter settings, the separation could be made to be substantially more fine grained, separately treating the “density islands” inside and around the two largest groupings. Here, we are focusing on the largest, most apparent separation and the two most populated clusters, colored in dark blue and gold. Coloring the UMAP based on the closeness centrality of the residue Ile126 (Figure 6c) gives a first indication of the conformational change causing the separation - it must involve a change in network topology that gives Ile126 a high closeness centrality in the right cluster. More clearly, the conformational change can be seen by constructing a “difference graph” between the two clusters.^10^ Here, this was done by calculating the consensus graphs for both clusters and subtracting one from the other. The resulting difference graph is shown as an arc diagram in Figure 6d. The inset (Figure 6e) shows the difference most clearly. There is a pair of neighboring isoleucines (Ile125 and Ile126) in a flexible loop of the CD, which swap places in their network connectivity. In either cluster, only one of the residues forms part of the hydrophobic network holding the domain core together, while the other points toward the solvent. In the right cluster (I126 in), Ile126 points to the domain core, leading to its increased closeness centrality, which is also visualized in the bar chart overlaid over the difference graph, which shows the difference in mean closeness fingerprints (Figure 6e). The bundles of exemplar structures in Figure 6b and the overlay of two exemplars in Figure S2d confirm the swap shown in the difference graph and illustrate the behavior of the flexible loop at the structure level. The two states are mutually exclusive and so are the topologies of their RINs. Averaging over them in constructing an RIN for the full ensemble would lead to an RIN topology that is physically impossible and not reflective of the residue interactions of FAT10. It is possible that the neighboring and alternating Ile residues equip the loop in the CD with additional flexibility while maintaining a stable fold. This illustrates the usefulness of analyzing local, side-chain-level conformational changes in residue interactions in a state-specific manner.

**Figure 6:**
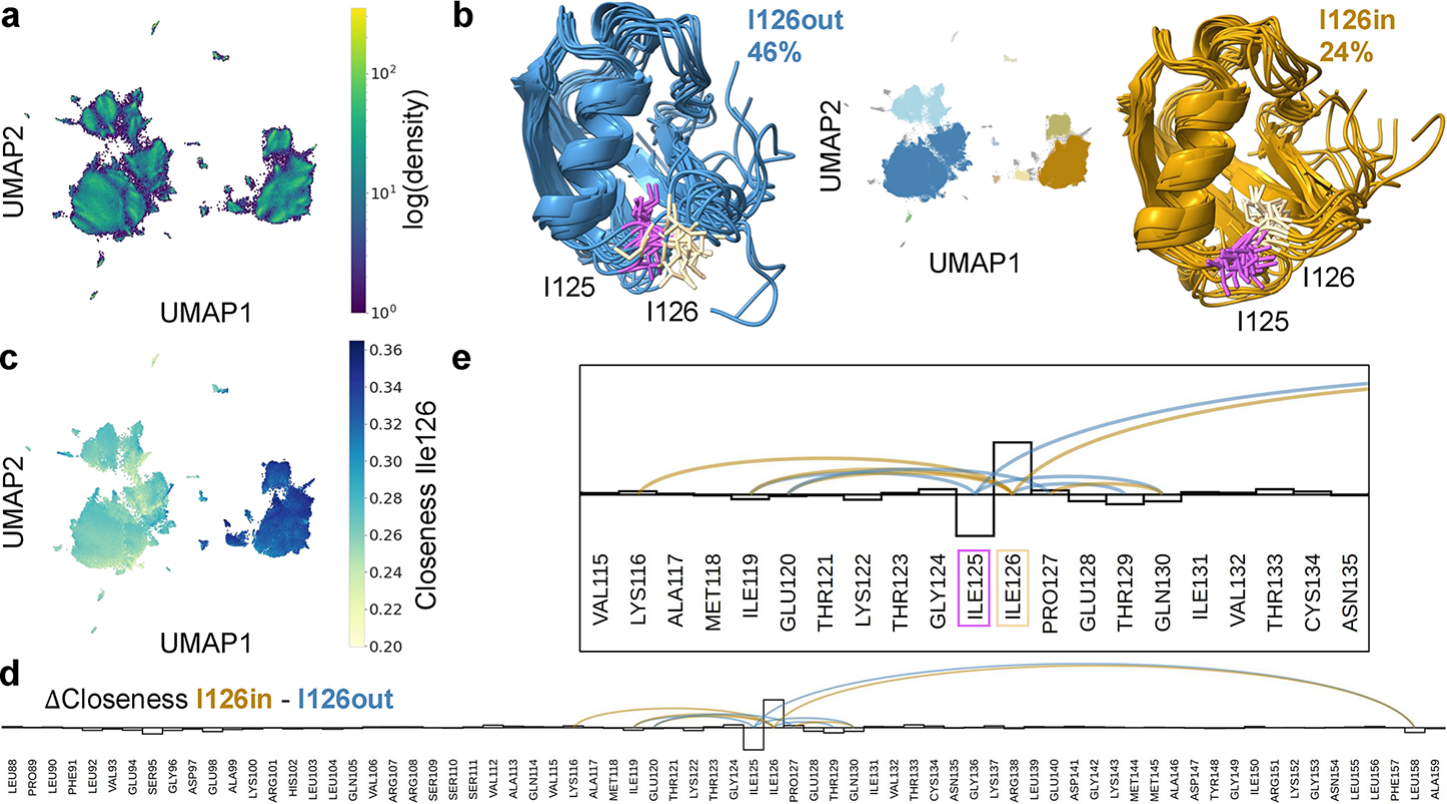
(a) UMAP of the closeness fingerprint of the CD in isolation, colored by log­(density). (b) UMAP colored by HDBSCAN cluster ID (inset) and representative cluster exemplars of the most populated HDBSCAN clusters of UMAP: I126 out (blue) and I126 in (gold). Residues Ile125 (lavender) and Ile126 (cream) are shown in stick representation. (c) UMAP colored by closeness centrality of residue Ile126. (d, e) Arc diagram of the difference graph between clusters Ile126out and Ile126 in. Arcs are drawn if there is an over 50% absolute difference of contact probabilities between both clusters and colored according to the sign of the difference to match cluster color. The difference in mean closeness fingerprints between the clusters is shown as a bar graph on top of the residues.

## Discussion and Conclusions

Expanding the toolkit of network methods by tools that can extract fine grained, state-specific network information from heterogeneous conformational ensembles is a crucial step in understanding the role of conformational heterogeneity and intrinsic disorder for protein function.^48,49^ Here, we have proposed a graph-based framework to identify residue interaction states from extensive MD simulations and perform state-specific characterizations. We employ the compact (*N*-dimensional) closeness centrality fingerprint to input RIN information from each simulation frame into off-the-shelf ML algorithms for dimensionality reduction and density-based clustering. The resulting residue interaction states (clusters of frames with similar RIN topology) are subsequently characterized at the different representation levels of the upstream workflow to elucidate relationships within and between them.

Applying this framework to a 30 μs MD data set of the two-domain protein FAT10, we have uncovered state-specific insight into FAT10s conformational behavior. Due to its fluctuating domain interactions and flexible regions, which are intimately tied to its function as a signaling protein^39,40^ it poses a formidable challenge to network analysis methods. Embedding the closeness fingerprint with a PCA gave a global, high-level overview of the diversity of domain interactions^39^ and their relative population in the residue interaction landscape. A more fine grained view was achieved with the nonlinear embedding by EncoderMap. Comparing two highly populated and adjacent states at the level of low-dimensional embedding and the closeness centralities showed that EncoderMap separated related, yet distinct residue interaction states. At the network level, the consensus graphs revealed the differences in contact topologies for both states in detail. Visualizing the prevalence of characteristic contact motifs for both states in the EncoderMap showed their role in the context of the full residue interaction landscape, elucidating how a local interaction in the flexible linker can influence the domain interactions globally. A UMAP of the closeness fingerprints of FAT10s globular CD revealed local residue interactions, showing how two residues in a flexible loop alternatingly participate in the interaction network of the hydrophobic domain core.

The characterization of clusters not only gives insight into the conformational ensemble of the protein, but it also allows an evaluation of the obtained clustering. This evaluation is essential, since there is no singular “true solution” or ground truth for unsupervised ML tasks like dimensionality reduction and clustering on a given data set. Similarly, there will be no one-size-fits-all workflow and parameter set for every system and downstream task. A different network generation formalism may allow the incorporation of more or less structural detail.^29,50,51^ While the closeness centrality captures RIN topology in an expressive and meaningful manner,^37^ other node-level representations of RINs might be suitable as well, such as other centralities^52^ or graph representation learning algorithms,^53^ possibly putting an emphasis on other features of the RIN description or including information beyond the network topology.^54^ Likewise, the choice of algorithms and parameters for dimensionality reduction and clustering will affect the analysis in terms of resolution, number of states and percentage of classified frames. Hence, visualizing each step of the processing pipeline and using it to gain insight into the simulation data ensures that the clustering result is useful to a practitioner trying to determine processing parameters appropriate for their task. The modularity of the framework and the compactness and intrinsic interpretability of the closeness fingerprint make it possible to flexibly and interactively adapt the workflow to the system at hand.

The framework offers a transparent and accessible platform for hypothesis generation and for making an informed selection of representative subsets of extensive MD data sets for downstream network analyses. On the level of the protein structures, such an informed subsetting can aid in network analyses where only a limited number of frames can be processed due to computational constraints.^5,50^ On the level of the RINs, it can also support applications that profit from state-specific resolution of network topologies. By constructing consensus graphs to represent individual states from the ensemble, one can retain information that may be blurred or lost when analyzing aggregated or averaged network descriptions of a full ensemble. Examples of this include the state-specific construction of elastic network models^55^ or investigations of allostery, such as identifying state-specific allosteric hubs,^56^ pathways^11^ or communities.^9^ By incorporating a low-dimensional visualization of the residue interaction landscape, the framework also allows a direct comparison of how the residue interactions change for conformational ensembles obtained at different conditions, e.g. with or without an allosteric effector,^57^ assessing the impact of such a modulation at every representation level, down to the RIN topologies of individual states and their relative weights. In this way, it is suited to provide a unified picture of an ensemble view of allostery and a network pathway view.^58^ It expands the toolbox of network methods to bring flexible protein systems and IDRs under the lens of state-specific network analysis.

## Methods and Computational Details

### Molecular Dynamics Simulations

The MD simulation data of FAT10 (Human leukocyte antigen (HLA)-F adjacent transcript 10) was produced by unbiased, atomistic simulation of the protein in full length (165 residues). 3 × 50 simulations were performed for 200 ns with a time step of 2 fs. For each simulation, 2001 frames at a temporal resolution of 100 ps/frame were analyzed, resulting in a data set of 300150 simulation frames. There were 2 × 25 different conditions for FAT10: 2 different ion concentrations (no additional ions and 150 mmol NaCl - physiological NaCl concentration) and 25 different starting conformations (domains fully separate and 25 different relative domain orientations). The MD simulations for FAT10 were carried out using the GROMACS simulation package,^59^ the GROMOS96 54/A7 force field^60^ and the SPC/E water model^61^ at constant temperature (300 K) and constant pressure (1 bar) conditions. Further details on the used simulation settings and the equilibration protocol can be found in Franke and Peter.^37^

### Processing Workflow

#### RIN Construction and Closeness Fingerprint Calculation

In the presented framework, the proteins’s atomic coordinates from each simulation frame are translated into a residue interaction network (RIN). In the RIN, each of the *N* amino acid residues is represented by a node. Two nodes are connected by an unweighted edge based on a geometric distance criterion. That means that the graph of the RIN is described by an *N* × *N* adjacency matrix *A*_*ij*_, where an entry for a node pair is 1 if the closest side chain distance (as calculated by the function compute-contacts with the side chain distance criterion in the python package mdtraj^62^) is below a cutoff of 4.5 Å or if they are direct neighbors in the sequence and 0 otherwise. By including the backbone into the contact criterion, we ensure that the graph is connected in any scenario, avoiding an unconnected (i.e., ill-defined) graph for calculating the closeness centralities. For this graph, the closeness centrality *c*_*i*_ of each node *v*_*i*_ is given by the reciprocal of the mean shortest path length *d*(*v*_*i*_, *v*_*j*_) from that node to all other nodes *v*_*i*≠*j*_, normalized by the number of nodes *N*:

$$c_{i}=\frac{N}{\underset{j}{\sum }d\left(v_{i},v_{j}\right)}$$

Since the edges of the graph are unweighted, the shortest (geodesic) path length *d*(*v*_*i*_, *v*_*j*_) is an integer count of the lowest number of edges separating *v*_*i*_ and *v*_*j*_. The method of RIN construction is a free parameter in the presented framework and should be chosen with care.^63^ The graphs were constructed using the python package NetworkX.^64^ The closeness centrality was calculated using the python package NetworKit.^65^ The *N*-dimensional vector of the *N* closeness centralities *c*_*i*_ - ordered by the amino acid sequence of the protein - then forms the closeness fingerprint describing each protein conformation from the simulation trajectory. It was transformed to be in a range of 0–1 by dividing by its maximum over all simulation frames. Further details on the calculation and on features of the closeness fingerprint can be found in Franke and Peter.^37^ For the analysis of the CD in isolation, the construction of the RIN was done analogously to full length FAT10, but considering only the contacts within the domain (residues 88 to 159).

### Dimensionality Reduction

#### Principal Component Analysis

Principal component analysis (PCA) is among the most commonly used dimensionality reduction schemes. It produces a linear transformation of the data set such that the directions (Principal Components/PCs) of the new coordinate system form an orthogonal basis with maximal variance along its first axes. By only taking the first *n* PCs of the transformation, one can effectively reduce the dimensionality of the data at hand to *n*, while retaining most of the variance of the data set. PCA is a linear dimensionality reduction method which best captures global features of the data. Here, the implementation of PCA in the python package scikit-learn^66^ was applied to the closeness fingerprint of full length FAT10. For the embedding, the first two PCs were used.

#### EncoderMap

EncoderMap^43,44^ is a neural-network-based nonlinear dimensionality reduction algorithm that combines a sketchmap-based distance cost with an autoencoder. EncoderMap can perform multidimensional scaling (MDS)-like dimensionality reduction based on the pairwise distance between data points. It has an autoencoder cost function, which reflects the difference between the input and the output, and an additional pairwise distance cost function, which forces the autoencoder to arrange the points in the low-dimensional map so that the arrangement of points in low-dimensional space reflects the distances between the points in high-dimensional space. The sigmoidal pairwise distance cost function used here was introduced in the MDS-like algorithm sketchmap^67^ and adapted for EncoderMap. It suppresses the impact of small distances between very similar points and of very large distances that would be hard to reproduce accurately in low-dimensional space. Details on parameter selection for EncoderMap and the sigmoid cost function can be found in Lemke and Peter^44^ and Ceriotti et al.^67^ The parameters used in applying EncoderMap to the closeness fingerprint of full-length FAT10 are described in Table 1.

**Table 1:** Parameters for EncoderMap

parameter type	parameter value
learning rate	0.00001
regularization const.	0.00001
periodicity	*∞*
*N*_frames_	300,150
*N*_steps_	25,000
sigmoid parameters	
σ_h_, *a*_h_, *b*_h_, σ_l_, *a*_l_, *b*_l_	1.1, 6, 6, 1, 2, 6

#### UMAP

Uniform Manifold Approximation and Projection (UMAP)^45^ is a nonlinear dimensionality reduction algorithm that produces a low-dimensional embedding of a high-dimensional manifold, attempting to preserve its topological structure or local neighborhood connectivity. It thus does not necessarily preserve global structure. UMAP was applied to the closeness fingerprint of the CD in isolation, in its python implementation with default parameter settings.^45^

#### Clustering

The clustering for all low-dimensional embeddings was performed using HDBSCAN (Hierarchical Density-Based Spatial Clustering for Applications with Noise).^46^ This clustering algorithm detects dense regions in the embedding and assigns each frame to either a cluster or to noise. It has been previously applied to MD data and has proven to be a powerful method to identify high-density regions in maps of conformational landscapes of proteins, i.e. (meta-)­stable conformational states.^28,36^ For the present analysis, the python package HDBSCAN was used with the parameters stated in Table 2. If not otherwise stated, other parameters were left at their default settings.

**Table 2:** Parameters for HDBSCAN

dimensionality reduction	min_cluster_size	min_samples	cluster_selection_method
PCA	100	800	“eom” (default)
EncoderMap	100	1200	“leaf”
UMAP isolated CD	1500	1500	“eom” (default)

### Cluster Representations

Since clusters from a large MD data set can contain several thousand members, some way of appropriately representing the cluster for the analysis task at hand and the representation level of the workflow must be established. At the level of the residue interaction landscape, the clusters were represented either by plotting the outlines of the clusters using the function kdeplot from the python package seaborn^68^ on top of the landscape or by coloring each point in the landscape by its cluster ID. At the node feature level, mean centrality fingerprints were calculated by taking the mean closeness centrality for each residue across all cluster members. At the level of the RIN, the clusters were represented by consensus graphs.^9^ The consensus graphs for the red and peach clusters from the EncoderMap were constructed by calculating the mean adjacency matrix across all cluster members. The probability for each contact in the cluster (a value between 0 and 1) is then used as an edge weight for this contact in the consensus graph. The contact probabilities were grouped into three categories (25–50%, 50–75%, >75%) for visualization. Differences between the clusters in the UMAP embedding were determined via a difference graph, which was constructed by calculating the difference between the two consensus graphs for both clusters. Both consensus and difference graphs were visualized using arc diagrams. The arc diagrams were drawn using the R^69^ packages ggraph^70^ and ggplot2 ^71^ and the R to python interface rpy2.

At the level of atomic coordinates or structures, a subset of the simulation frames was selected to represent a cluster. Three different strategies for this are presented here. For representative structure bundles, 10 frames were selected, evenly spaced from the cluster member list. This was done for the clusters found in the PCA embedding of full-length FAT10 and results in a frame selection that represents the conformational diversity of the clusters well.

For exemplar structure bundles, the function “exemplars” from the package HDBSCAN^46^ was used. This function returns a number of the “most representative” points for each cluster. From these, 10 evenly spaced frames were selected as the final exemplars. This results in a bundle of the most characteristic frames for a cluster. This strategy was applied for the EncoderMap of full-length FAT10 and for the UMAP of the CD.

Where individual structures are shown, they were chosen by calculating the centroid of the exemplars - picking the one “centroid” structure that was closest to all other exemplars based on their closeness fingerprints. The preselection via the exemplar function was useful to obtain characteristic structures for the clusters and at the same time save computational time when selecting the centroid.

### Data Analysis and Visualization

All data analysis tasks were performed using Python 3. General computations were performed using NumPy^72^ and pandas.^73^ Structural analyses, such as calculations of *R*_g_ and distances were performed using MDTraj.^62^ The contacts between FAT10s domains were calculated by separating the ND into two sections (red and blue) between residue Val43 and Pro44. and summing up the number of contacts - determined by the distance criterion described above - of the CD with the respective sections of the ND. The two contact counts were combined by counting the contacts with the blue section negatively. Data visualization was performed using Matplotlib.^74^ Visualization of molecular structures was performed using ChimeraX.^75,76^

## Supplementary Material



## Data Availability

The FAT10 data set underlying this study is available in the research data repository KonDATA at the following link: https://doi.org/10.48606/gx4q9ureeuzxsndv. The Python code for the analysis framework is available at: https://github.com/AG-Peter/Clustering_Networks.

## Notes

Overlays of representative structures for clusters from PCA, EncoderMap, and UMAP, PCA plots with first three principal components, series of EncoderMaps colored according to the closeness centralities of individual residues, and time trace of individual trajectories mapped onto EncoderMap (PDF)
