# Supplementary material for: Clustering and Analyzing Ensembles of Residue Interaction Networks from Molecular Dynamics Simulations
Source: J Chem Inf Model. 2025 Oct 3;65(20):11203–14. doi: 10.1021/acs.jcim.5c01298 (PMC12570133; doi:10.1021/acs.jcim.5c01298)
Supplement: Supplementary file 1 [file ci5c01298_si_001.pdf]

SUPPORTING INFORMATION: Clustering and Analyzing Ensembles of Residue Interaction Networks from Molecular Dynamics Simulations

Leon Franke* and Christine Peter*

Department of Chemistry, University of Konstanz, Konstanz 78457, Germany

E-mail: Leon.Franke@uni-konstanz.de; Christine.Peter@uni-konstanz.de

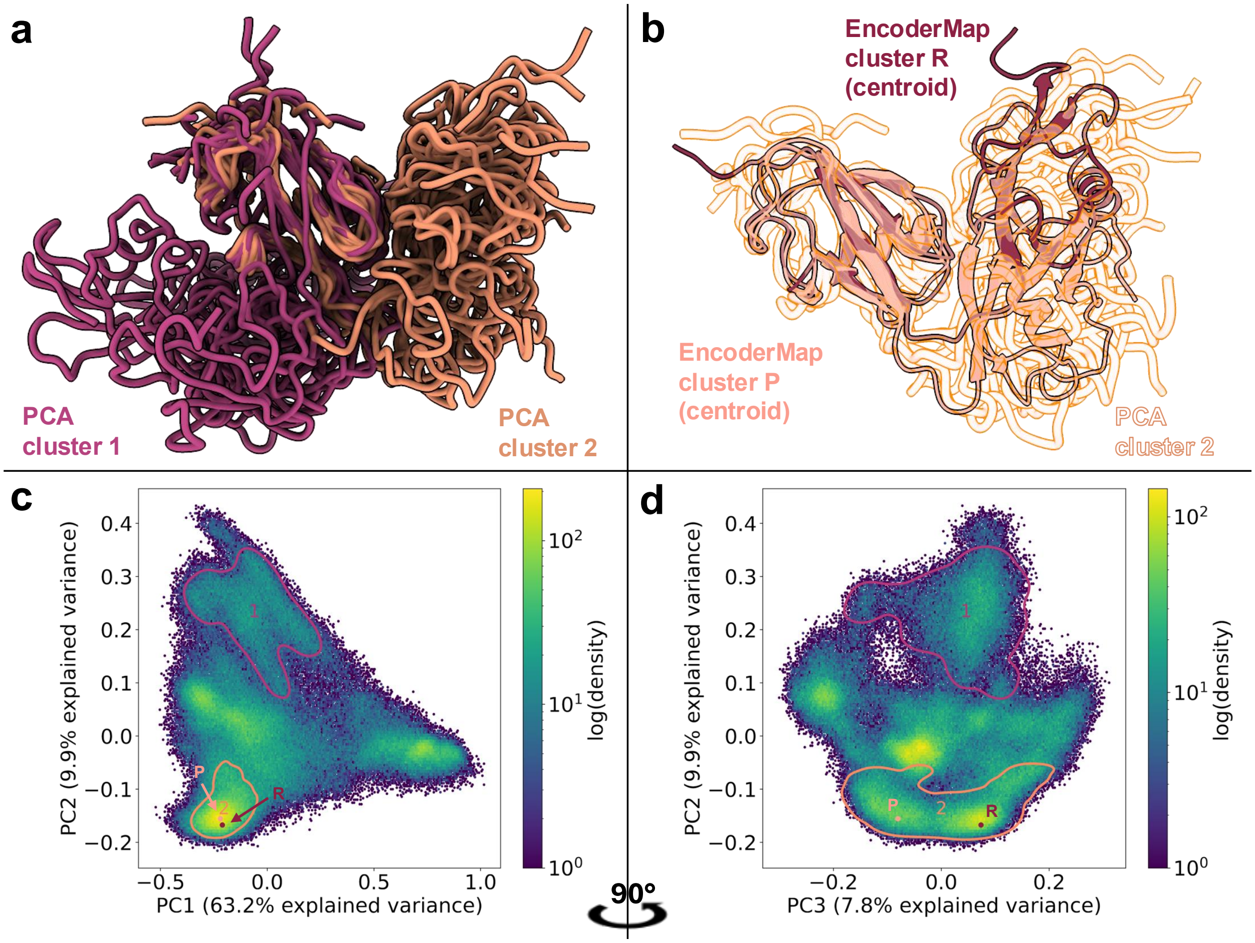

Figure S1: **a** Overlay of 10 exemplars of cluster 1 (purple) and cluster 2 (orange) of the PCA, aligned on the N-terminal domain, drawn in licorice style. **b** Overlay of 10 exemplars of PCA cluster 2 (licorice with orange outlines) and the centroids of EncoderMap clusters P (peach cartoon) and R (red cartoon), aligned on the N-terminal domain. **c** PCA (PCs 1 and 2), with outlines of PCA clusters 1 and 2 and the positions of the centroids of EncoderMap clusters P (peach dot) and R (red dot). **d** PCA (PCs 3 and 2), with outlines of PCA clusters 1 and 2 and the positions of the centroids of EncoderMap clusters P (peach dot) and R (red dot). It can be seen that PCA cluster 2 contains P and R from the EncoderMap.

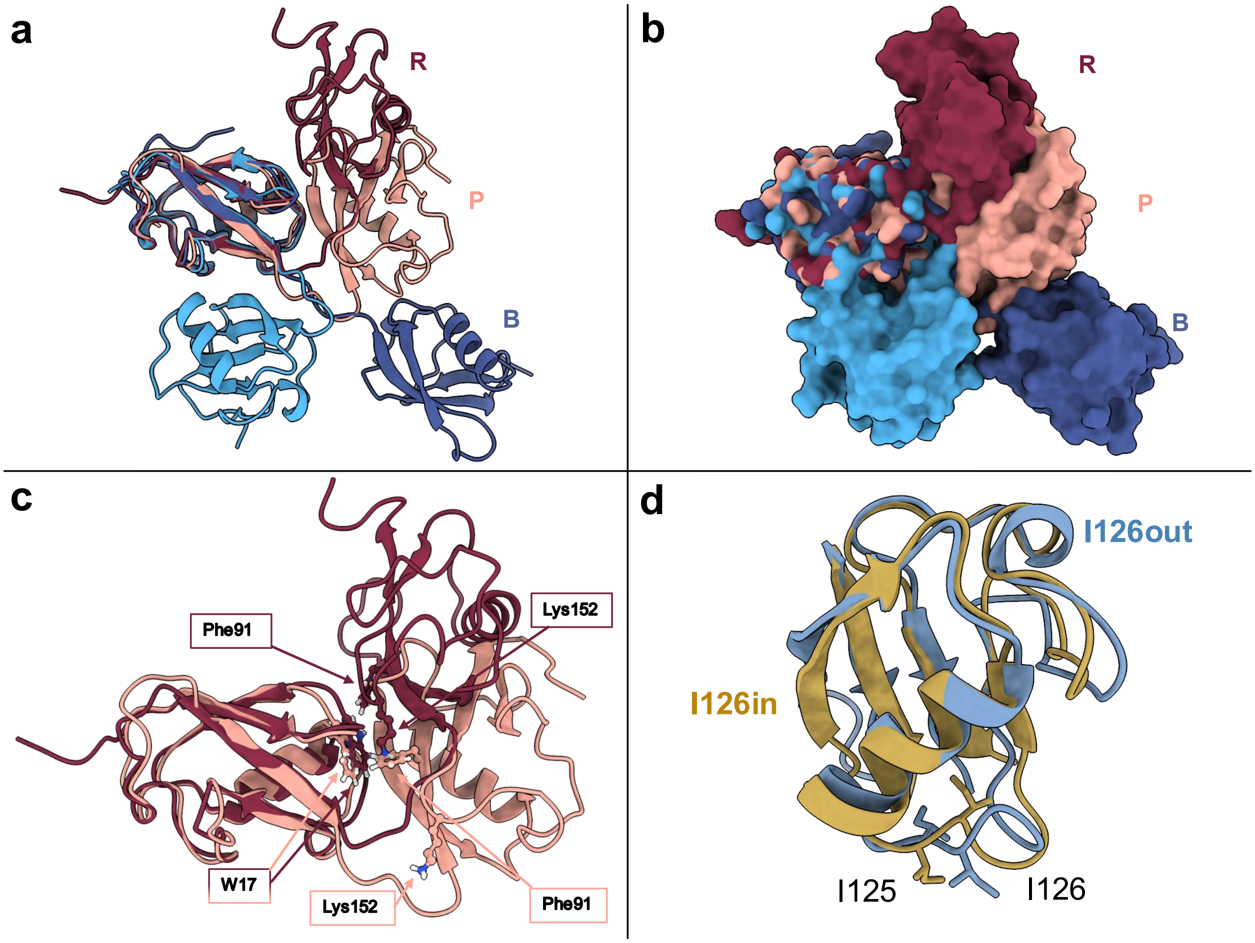

Figure S2: Overlay of the centroids of the 4 most populated EncoderMap clusters: R (red), P (peach), B - open cluster (dark blue), 4th most populated cluster (light blue), drawn in cartoon **a**, and as surfaces **b**. **c** Overlay of centroids of R (red, cartoon) and P (peach, cartoon), with some interface residues with high closeness centralities drawn as ball and stick. **d** Overlay two structures from the most populated UMAP clusters I126in (gold cartoon) and I126out (blue cartoon), with the Ile residues shown as sticks.

Figure S3a-c: Series of EncoderMaps colored according to the closeness centralities of individual residues

The changes of the closeness centrality of an individual residue across the low-dimensional map of the residue interaction landscape can help to understand the role of that residue for the protein structure and dynamics and to identify residues that merit a closer inspection. If a residue displays strong pattering across the landscape, having very high closeness values in some regions and relatively low values in others, this indicates that this is a residue taking part in a domain interface in some states but not in others (e.g. TRP17 or ARG60 in Figure S3**a**). On the other hand, if a hydrophobic residue displays only a general increase of closeness in correlation with the closing of the protein and shows very little pattering across the map otherwise, this can be an indication that it does not participate in a specific interface and it could be part of the hydrophobic core of a domain (e.g. ILE33 in Figure S3**a** or VAL132 in Figure S3**c**). Another example are the intrinsically disordered terminal tails (residues MET1-CYS7 (Figure S3**a**) and TYR161-GLY165 (Figure S3**c**), which are marked by a fairly low closeness centralities across the full residue interaction landscape, because they move relatively rapidly. If there are regions in the map that do display a higher centrality for the tail-residues, the tail is likely to form a relatively stable contact pattern with the rest of the molecule, possibly taking part in the domain interface. Both tails display these regions of relatively high closeness at the high-contact fringes of the map. The residues in the intrinsically disordered linker (LYS82-GLU86 (Figure S3**b**)) are marked by a relatively even and intermediate closeness across the landscape. While this overview can give some insights on the level of individual residues, these tendencies should be evaluated at other levels of the workflow as well, e.g. the contact network level or the structure level.

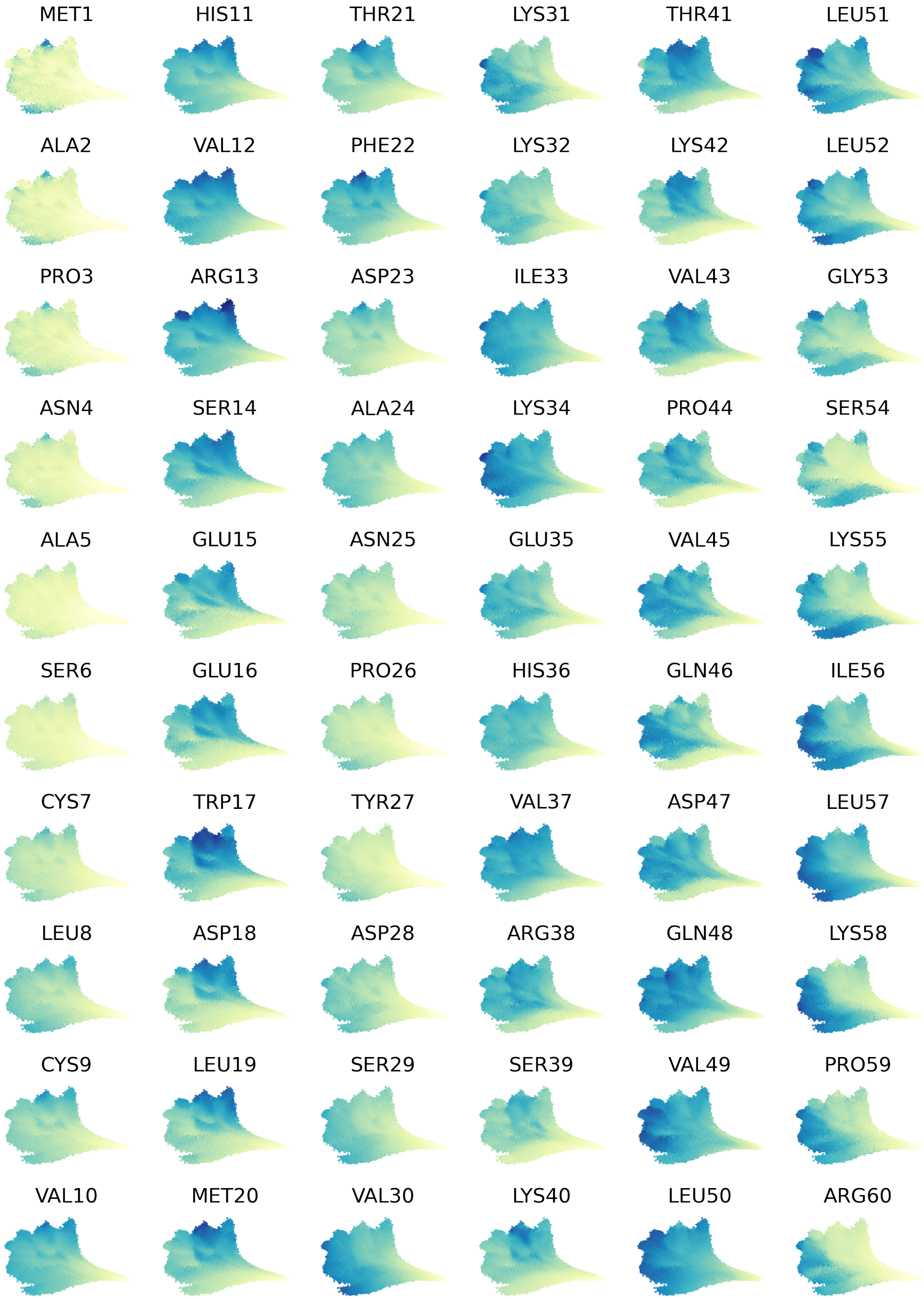

Figure S3**a**: EncoderMap colored by closeness centralities for MET1-ARG60

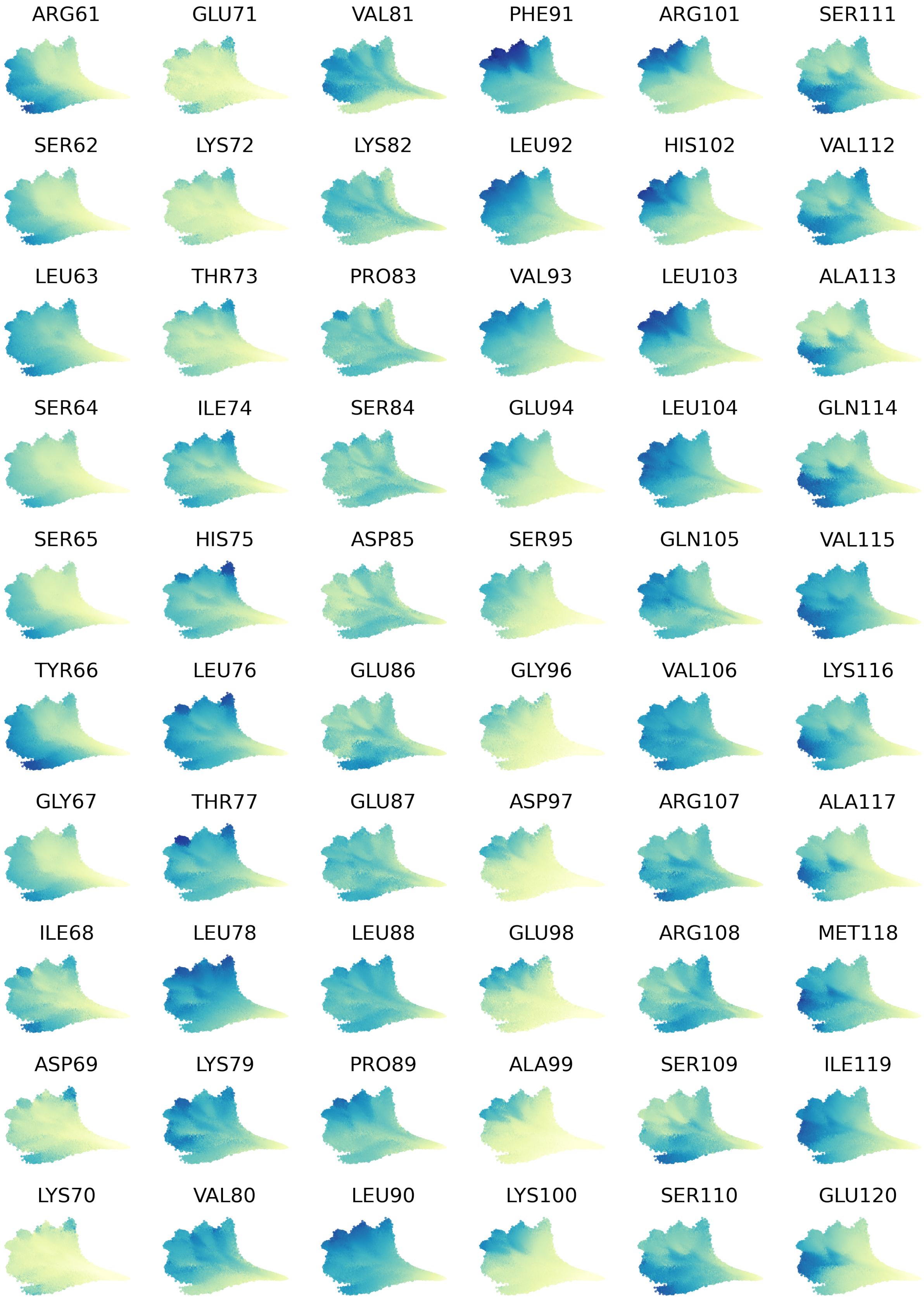

Figure S3**b**: EncoderMap colored by closeness centralities of ARG61-GLU120

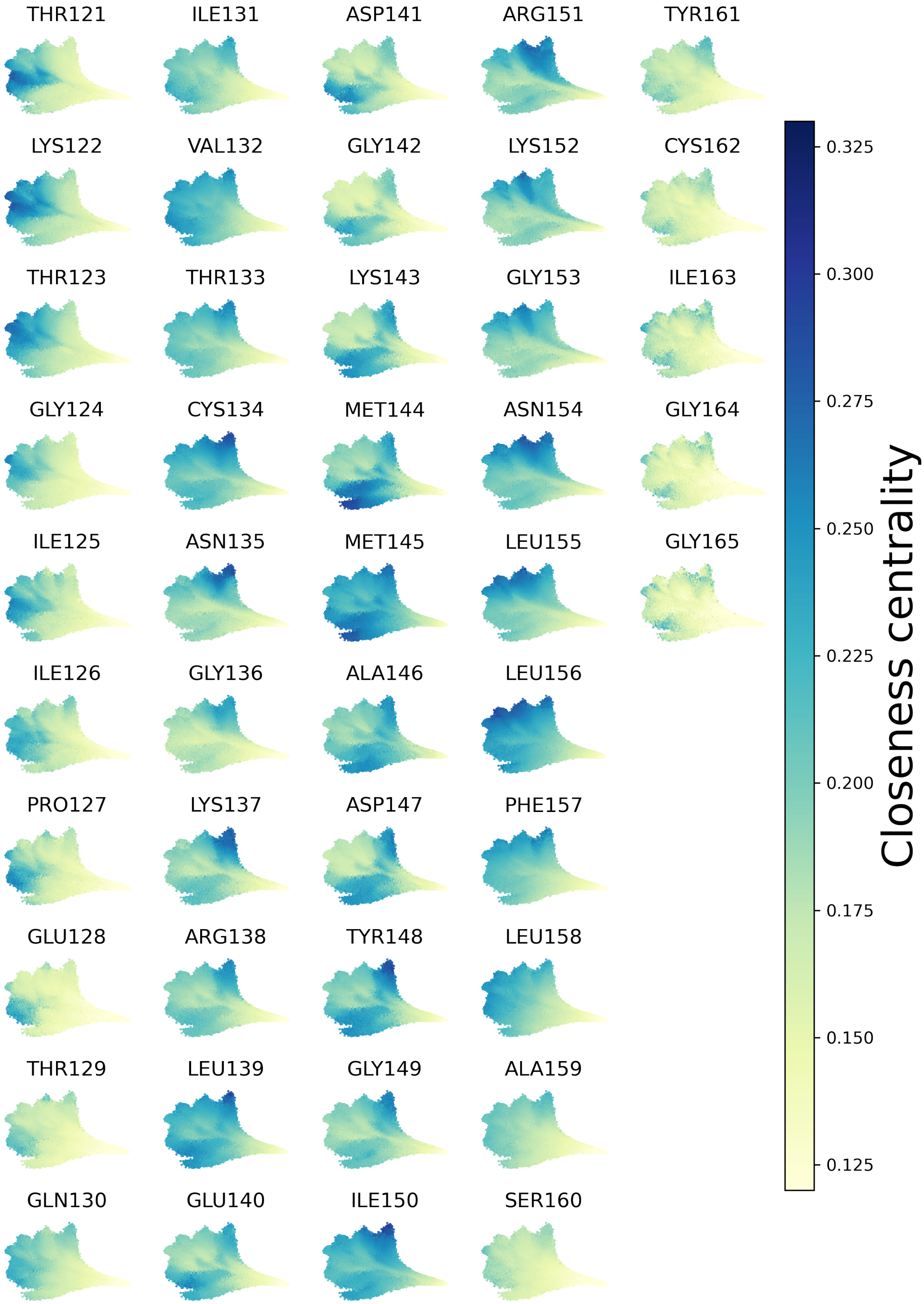

Figure S3**c**: EncoderMap colored by closeness centralities of THR121-GLY165

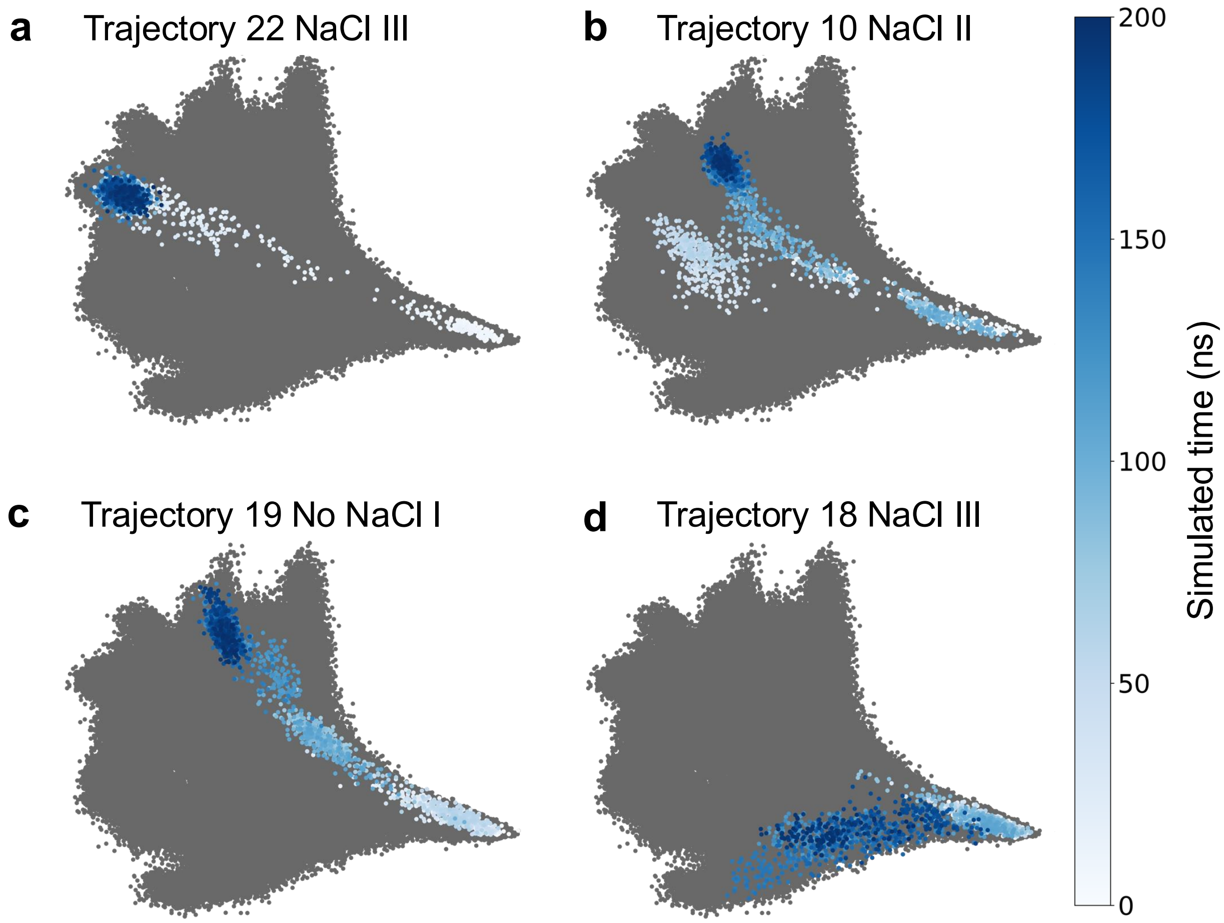

Figure S4: Time trace of individual trajectories (200 ns, darker blue means later time) mapped onto the EncoderMap (grey), in which FAT10 (**a**) closes quickly and finds a stable closed state, (**b**) closes to a less stable state, reopens fully and then moves to a stable state, (**c**) closes more slowly via an intermediate, less closed state, or (**d**) explores a relatively shallow region of the residue interaction landscape without closing into a stable state.
